# Ten misconceptions about trauma-focused CBT for PTSD

**DOI:** 10.1017/S1754470X22000307

**Published:** 2022-07-22

**Authors:** Hannah Murray, Nick Grey, Emma Warnock-Parkes, Alice Kerr, Jennifer Wild, David M. Clark, Anke Ehlers

**Affiliations:** 1 Department of Experimental Psychology, University of Oxford, Oxford, United Kingdom; 2 Oxford Health NHS Foundation Trust, Oxford, United Kingdom; 3 Sussex Partnership NHS Foundation Trust, United Kingdom; 4 University of Sussex, United Kingdom; 5 King’s College London, London, United Kingdom

**Keywords:** CBT, PTSD, training, cognitive therapy

## Abstract

Therapist cognitions about trauma-focused psychological therapies can affect our implementation of evidence-based therapies for posttraumatic stress disorder (PTSD), potentially reducing their effectiveness. Based on observations gleaned from teaching and supervising one of these treatments, cognitive therapy for PTSD (CT-PTSD), ten common ‘misconceptions’ were identified. These included misconceptions about the suitability of the treatment for some types of trauma and/or emotions, the need for stabilisation prior to memory work, the danger of ‘retraumatising’ patients with memory-focused work, the risks of using memory-focused techniques with patients who dissociate, the remote use of trauma-focused techniques, and the perception of trauma-focused CBT as inflexible. In this article, these misconceptions are analysed in light of existing evidence and guidance is provided on using trauma-focused CT-PTSD with a broad range of presentations.

Trauma-focused cognitive behavioural therapies such as Cognitive Therapy for PTSD (CT-PTSD, Ehlers & Clark, 2000), Prolonged Exposure (Foa & Rothbaum, 1998) or Cognitive Processing Therapy (Resick & Schnicke, 1992) are very effective in treating posttraumatic stress disorder (PTSD) and are recommended as first-line treatments in NICE (National Institute for Health and Care Excellence, 2018) and international guidelines (e.g. International Society for Traumatic Stress Studies, 2019a). However, a research-practice gap exists between the efficacy of these treatments achieved in clinical trials and the effectiveness of the treatments delivered in routine clinical practice (see Foa et al., 2013). For example, *Improving Access to Psychological Therapies* (IAPT) services currently report that on average around 40% of PTSD patients meet IAPT criteria for recovery at discharge, which includes recovery on self-report measures of both PTSD and depression (NHS Digital, 2021). While this is a strict criterion for recovery, it is likely to reflect a lower effectiveness than that reported in the research literature on PTSD and is certainly below the IAPT target of *at least* 50% recovery which was based on a consideration of the available literature from RCTs (Layard et al., 2007).

This is not a unique problem to PTSD treatments. Often something gets lost in translation from the research clinic to the therapy room, for various reasons. Clinicians working in routine clinical services are likely to be less highly trained in a particular treatment model and less closely supervised than trial therapists. The IAPT high intensity training course, for example, currently only includes two days of teaching on PTSD plus a requirement to treat one relevant case, with no minimum amount of supervision specified. Therapists may also face local organisational constraints, such as shorter and fewer sessions and difficulty scheduling out of office sessions for behavioural experiments or revisiting the site of the trauma. Patients in clinical trials have to meet certain inclusion and exclusion criteria so may be arguably less ‘complex’ than those seen in routine practice.

However, if we take the example of CT-PTSD, most trials have had relatively few exclusion criteria. Furthermore, dissemination trials with no exclusions (Duffy et al., 2007) and routine clinical practice audits of specialist clinics (Ehlers et al., 2013; Gillespie et al, 2002) have found CT-PTSD to be a highly effective treatment in routine clinical care.

Another possibility is that some therapists may miss out effective elements of trauma-focused treatments for various reasons, particularly addressing trauma memories, out of office behavioural experiments and site visits, especially with patients deemed complex, leading to poorer treatment outcomes. For example, in an effectiveness study of CT-PTSD in routine clinical care, Ehlers et al. (2013) found that treatment was less trauma-focused for patients with multiple traumas and social problems, and more of their sessions dealt with non-trauma-related problems compared to other patients. This may be due in part to the necessity of responding to crises in the patients’ everyday life, but may also reflect therapists’ concerns. Therapist cognitions may interfere with the adoption of trauma-focused techniques, driven by worries about ‘retraumatising’ patients, or simply not having the experience or confidence in using certain treatment strategies.

A number of studies have found that, despite positive attitudes towards trauma-focused treatments, many clinicians do not fully deliver the treatment, particularly exposure-related tasks (e.g. Becker et al., 2004; Borah et al., 2017; Gray et al., 2007; van Minnen et al., 2010). This has been linked to beliefs that exposure therapy is intolerable, unethical and unsafe (Deacon & Farrell, 2013); therapists scoring highly on such beliefs show a more cautious approach to delivering treatment (Deacon et al., 2013). The most common barriers to delivering evidence-based treatments for PTSD cited are perceived inflexibility of manualised approaches, fear of increasing patient distress, working with comorbidities and a lack of training and support (Finch et al., 2020). Service-related factors, such as shorter and fewer sessions than recommended on the basis of the evidence, may feed into therapist cognitions about the suitability and applicability of TF-CBTs with their patients.

Over many years of training and supervising therapists to use CT-PTSD, we have repeatedly encountered certain misconceptions about trauma-focused therapies. For example, through a Health Education England-funded nationwide top-up training and supervision programme in CT-PTSD which has, so far, trained around 300 therapists, a study of internet-delivered CT-PTSD in IAPT services, and through numerous other training and supervision experiences, we have received feedback from therapists and supervisors about implementing CT-PTSD in clinical practice. From this, we have derived ten misconceptions which seem to be commonplace, which we discuss in this paper. Most of these apply to all trauma-focused psychological therapies, although we give examples drawn from our own practice, which is in CT-PTSD. The final two are more specific to CT-PTSD.

In addressing these misconceptions, we do not intend to shame or discourage any therapists who have believed them. We have many similar thoughts ourselves at times and have had the opportunity to test them out. Having trained and supervised hundreds of therapists, we have huge respect and admiration for their skill, effort and dedication to their patients. In fact, many of these misconceptions stem from a compassionate urge to protect our patients from further distress. Others seem to arise from a lack of confidence, limited experience of particular presentations or techniques, inadequate training opportunities, or have been perpetuated by well-intentioned supervisors or trainers, who have themselves heard them elsewhere and passed them on. We all (ourselves included) ‘drift’ from evidence-based treatments at times (Waller, 2009), influenced by our beliefs, emotions, experiences and personalities. Waller and Turner (2016) suggest therapists use CBT on ourselves: self-monitoring, restructuring our beliefs and testing them through behavioural experiments. We agree, and offer this article as psychoeducation and a prompt for self-reflection.

## CT-PTSD

There are a number of TF-CBTs which share many features (such as a focus on processing trauma memories) but certain differences in approach and techniques. Here we describe CT-PTSD, one form of TF-CBT, as this is the treatment approach we refer to throughout the paper. CT-PTSD is based on Ehlers and Clark’s (2000) cognitive model of PTSD which suggests that PTSD is maintained via three processes:

- *Meanings* that arise from the way an individual has appraised the traumatic event or its aftermath which lead to an ongoing sense of threat. This may be external threat (e.g. that the world is more dangerous than they previously realised) or internal threat to the self (e.g. the trauma showing that they are a bad, inferior or damaged person).
- *The nature of trauma memories*. Due to the way that a trauma has been processed (e.g. as a stream of sensory impressions, as unreal/not happening to the self), the worst moments of the trauma are poorly elaborated and disjointed from other autobiographical information in memory. These types of memories are easily triggered and have a ‘here and now’ quality when they are recalled; and people may be unable to access other information that could correct impressions or negative beliefs they had at the time, or make sense of their experiences.
- People with PTSD develop *cognitive and behavioural coping strategies* to reduce their sense of external or internal threat. These strategies can inadvertently increase symptoms (e.g. memory suppression or substance use) or the sense of threat (e.g. hypervigilance to danger). Importantly, avoidance, safety behaviours, social withdrawal, substance use and rumination prevent change (reappraisal) of traumatic meanings or in the nature of the trauma memory, which remains in its poorly elaborated, disjointed state.

To address these core processes, CT-PTSD aims to work on the problematic meanings associated with the trauma, process the trauma memories, and identify and drop maintaining cognitive or behavioural strategies. Core treatment procedure in CT-PTSD include:

- *Individualised case formulation –* the therapist and patient collaboratively develop an individualised formulation, which serves as the framework for therapy.
- *Reclaiming/rebuilding your life assignments* are planned from the first session onwards to address the patients’ perceived permanent change after trauma. They include re-starting activities and social contact that the patient has stopped since the trauma and/or beginning new activities, and are included as an agenda item in every session.
- *Changing problematic appraisals* of the trauma (both peri and post-traumatic) through a variety of cognitive techniques including guided discovery and behavioural experiments.
- *Updating trauma memories* is a three-step procedure that involves (i) accessing memories of the worst moments during the traumatic events (‘hotspots’) and their associated meanings, (ii) identifying information that updates these meanings (either information from the course of events during the trauma or from cognitive restructuring and testing of predictions), and (iii) linking the new meanings to the worst moments in the memory (‘updating’).
- *Stimulus discrimination training with triggers of reexperiencing* involves systematically spotting idiosyncratic triggers (often subtle sensory cues) and learning to discriminate between THEN (cue in the traumatic event) and NOW (similar cue in a new safe context).
- *A site visit* where possible for greater consolidation of memory updating and trigger discrimination.
- *Dropping unhelpful behaviours and cognitive processes* commonly includes discussing their advantages and disadvantages, and behavioural experiments where the patient experiments with reducing unhelpful strategies such as rumination, hypervigilance, thought suppression and excessive precautions (safety behaviours).
- A *blueprint* summarises what the patient has learned in treatment and includes plans for addressing any setbacks.

For more information and details on how to use CT-PTSD, including therapist training videos, see www.oxcadatresources.com (registration and unlimited use is free of charge).

## Misconceptions about TF-CBT

### 1 Trauma-focused treatments are not suitable for complex/multiple trauma

One of the most common misconceptions we hear is that TF-CBT is unsuitable for working with the experience of multiple or complex traumas. This observation is echoed in other studies, for example in van Minnen et al.’s (2010) finding that therapists perceive more barriers and are less likely to choose imaginal exposure for patients who have experienced multiple childhood traumas compared to a single incident trauma, particularly when they are also experiencing comorbid depression.

Existing evidence does not support this misconception. For example, CT-PTSD remains effective when patients have experienced multiple traumas, with only a slight decrease in symptom reduction compared to single incident trauma (Ehlers et al., 2013), although more time may be required in therapy. There is also evidence that a range of trauma-focused treatments are effective following childhood sexual abuse (Ehring et al., 2014) and for complex PTSD (a new diagnosis in ICD-11; World Health Organisation; 2018), although further research is needed in this emerging field (Karatzias et al., 2019). Treatment guidelines consistently recommend trauma-focused treatments, regardless of trauma type (e.g. American Psychological Association, 2017; ISTSS, 2019a; NICE, 2018). Additional considerations are recommended for multiple trauma, such as allowing for more sessions and addressing barriers to engaging with trauma-focused treatment like substance misuse (NICE, 2018), as well as for complex PTSD, for example the personalisation of treatment, including additional modules to address problems associated with complex PTSD (ISTSS, 2019b).

In CT-PTSD, there is a certain economy in addressing multiple traumas as the individual case formulation identifies links in meaning between different traumas (e.g., ‘no one can be trusted’, ‘I am worthless’, ‘people will look down on me if they knew what happened’), which can then be addressed together. Guided discovery leads to a reevaluation of these appraisals, which is then used to update the most relevant linked hotspot. The therapist then facilitates generalisation to related hotspots from other traumas. Similarly, the formulation identifies appraisals and behaviours that maintain PTSD and comorbid conditions such as depression or panic disorder, so that comorbidity can be addressed in an integrated and focused way (see case examples in Ehlers & Murray, 2020; Ehlers & Wild, 2020).

Therapists may also be concerned that trauma-focused treatments which involve reliving or narrative writing may not suitable for very long or repeated traumas because recounting the whole trauma stories would take too much time in treatment or would be too distressing. However, the updating memories procedure does not require all details from the trauma to be recounted as it focusses on the moments of the patients’ traumas that are reexperienced and give rise to important threatening meanings about the self and the world. Hotspots are updated one at a time once their meanings are explored, and those with meanings that are straightforward to update (e.g., ‘I did not die’, ‘I can still walk’, ‘my body has healed’) can be addressed early in treatment without necessarily eliciting all other parts of the respective trauma. The emotional shift and reduction of reexperiencing after updating a hotspot may then motivate patients to disclose more details about others, e.g. those linked to self-blame and shame.

### 2 Stabilisation is always needed before memory work

A linked appraisal is that a stabilisation phase of treatment is always needed prior to a trauma-focused intervention, especially for those with multiple traumas. This misconception perhaps originates from Herman’s (1992a) phased model for working with complex PTSD. She recommended that a stabilisation phase should precede a trauma-processing phase, followed by a phase of ‘reintegration’ with important areas of life such as relationships, socialising and work. However, the interpretation that these phases should be followed in a strict linear fashion is itself a misunderstanding. Herman (1992b) emphasised that the phases of recovery are “oscillating and dialectical in nature” and that “like any abstract concept these stages of recovery are a convenient fiction, not to be taken too literally” (p. 155).

A phase-based approach has high face validity and has been recommended in various expert consensus guidelines (e.g. McFetridge et al., 2017), and underlies effective treatment packages (e.g. Bohus et al., 2020; Cloitre et al., 2002). However, there is limited evidence that phase-based approaches are superior to interventions that primarily focus on trauma processing, with critics arguing that they are an unnecessary use of clinical time, neither improving outcomes nor reducing drop-out (Baekkalund et al, 2021; de Jongh et al., 2016; Oprel et al., 2021). There is also great variability in what constitutes stabilisation, what interventions are offered and whether this is provided individually or in a group. There is recently a move towards considering ‘multi-component interventions’ encouraging individualised rather than phase-based approaches (Coventry et al., 2020).

CT-PTSD does not include a formal stabilisation phase and work on trauma memories in the form of imaginal reliving, narrative writing or constructing a timeline usually begins in session two of treatment. Session one includes psychoeducation and formulation (see oxcadatresources.com for a video on the first session of treatment), building the foundations of a collaborative and safe therapeutic relationship.There are instances when other interventions are also used prior to memory work to make sure that the patient can process the trauma memories in a productive way. For example, we always address risk as a priority, including risk to self, others and from others (including the perpetrator), and often adjust the order of interventions depending on individual needs and the formulation (see also point 10). If someone is very dissociative, we introduce trigger discrimination prior to updating memories work, and usually work on a written narrative first rather than reliving; verbally describing the event and writing it down at a pace that allows awareness of the present situation (i.e. being with the therapist in their office, years after the trauma) to be maintained at all times. Where people experience high levels of shame or a sense of enduring degradation or defeat associated with the trauma, we often address the related appraisals prior to memory work and develop some preliminary updates which can be introduced into the trauma memory immediately during reliving or narrative writing. Where people have lost a loved one during the trauma, the therapist and patient would talk about the person they lost, their relationship and what the person meant to them before reliving the trauma. However, we do not view these interventions as a formal stabilisation phase, rather as individualisation of treatment dependent on needs, guided by formulation.

Some of our patients are experiencing instability in their life circumstances, such as an imminent house move, financial difficulties, treatment for medical problems or an upcoming court case. Patients may prefer to wait until they have resolved these issues before beginning treatment, but we generally proceed if they consent and feel able to engage with treatment. The latest Crown Prosecution Service guidance on pre-trial therapy (2020, currently under consultation) emphasises prioritising the wellbeing of the victim, giving informed choice regarding treatment, but not delaying therapy if it would be in their best interest. We have also noticed that some therapists delay treatment when symptoms are very severe and focus instead on symptom management. However, this can prevent patients receiving the effective elements of treatment which will have a greater impact on their symptoms. For example, sleep typically improves following work on the trauma memories (Woodward et al., 2017), so poor sleep should not be a reason to delay.

Overall, our experience is that most patients do not require a separate phase of stabilisation, and that additional interventions should facilitate work on updating trauma memories rather than replace them. Some of the interventions which form part of stabilisation packages may be included in CT-PTSD, typically interwoven with other interventions rather than as a distinct phase, and on a ‘needs-only’ bases. We recommend that treatment is individualised to specific needs rather than a ‘one size fits all’ approach. However, further empirical studies are needed on whether some patients with complex PTSD benefit from stabilisation prior to trauma-focused work and how such interventions might be best offered. We also do not believe that reintegration should wait until the end of treatment; in CT-PTSD, ‘reclaiming/rebuilding your life’ starts in session one and is part of every session, and contributes to changing appraisals of permanent change (e.g. ‘I am permanently changed for the worse’) and feelings of hopelessness.

### 3 Talking about trauma memories is ‘retraumatising’

The term ‘retraumatisation’ is used in different ways. Strictly, it means being exposed to a further traumatic experience but clinicians commonly use it in the context of fearing that deliberately thinking about memories of past traumatic events is itself traumatic, and will cause symptom worsening. Undeniably, many of our patients become distressed when talking about their trauma memories, and some (not all) experience a temporary modest symptom exacerbation afterwards, so it is understandable that an empathic therapist may fear that they are somehow harming their patients. Yet, we know from research that engaging with, rather than avoiding, trauma memories through talking and/or writing is one of the most effective elements of treatment and that patients interviewed after reliving have reported it is ‘worth the pain’ (Shearing et al., 2011). We also know from randomised controlled trials that more patients experience a deterioration of symptoms in waitlist conditions than in trauma-focused treatments (e.g. Ehlers et al., 2014; Jayawickreme et al., 2014). Similarly, studies of patients treated in routine clinical care show that few people deteriorate over a course of trauma-focused CBT (e.g. 1.2% in Ehlers et al., 2013; 0% in Gillespie et al., 2002), and it remains unclear whether this is due to treatment or new life events/stressors.

Introducing and managing work on trauma memories carefully is important in minimising any negative impact. Patients should be given information about how PTSD is maintained, including the role of unprocessed memories and avoidance, to give a clear rationale for why talking about memories can be helpful, so that they can give informed consent. Metaphors are particularly useful here. Work on trauma memories is conducted within a strong, collaborative therapeutic relationship where the patient feels in control of the process, supported by the therapist and understands confidentiality. The therapist prepares the patient for the possibility of temporary distress and symptom exacerbation and helps them plan for after the session, for example, support from a loved one or a pleasurable activity. As well as the important processing of trauma memories, revisiting the trauma in imaginal reliving or narrative writing within these parameters also sends positive messages: that the trauma memories, associated meanings and distress are tolerable, that the patient can take control over the memories rather than be controlled by them and that the therapist will accept and appreciate the patient no matter what has happened. Since people with PTSD already re-experience the past traumatic events in flashbacks and nightmares, in treatment we are simply asking them to engage with these memories in a more structured and supported way.

### 4 Some traumas shouldn’t be relived

Therapists sometimes express anxieties about working with certain types of trauma. We have heard misconceptions that imaginal reliving should not be used with sexual assault memories (even though Foa’s early work and trials on Prolonged Exposure therapy as well as Resick’s early work on Cognitive Processing Therapy focused on women who developed PTSD after rape, e.g. Foa & Rothbaum, 1998; Foa et al., 2005; Resick & Schnicke, 1992; Resick et al., 2002), traumatic bereavements, childhood traumas, if there are gaps in the memories or a patient had lost consciousness, following multiple or very long traumas, where the patient’s worst fear had come true (e.g. someone died in the trauma), or where the trauma was a patient’s fault.

Our experience is that PTSD associated with all types of trauma can be addressed with CT-PTSD and that reliving or a written narrative is nearly always possible. Some adaptations may be required. For example, memories which are disjointed or contain a lot of gaps can benefit from being mapped out with a timeline first. Imaginal reliving of the parts of the memory which can be recalled can still be helpful, although written narratives can be easier if the memory is very fragmented. Finding information to explain gaps and to access a plausible scenario for what occurred can be helpful, for example using reports from others or an early site visit. For example, patients traumatised by treatment in intensive care units (ICU) typically have incomplete and confused memories due to periods of sedation. They often find ICU diaries, medical records and accounts from loved ones useful in understanding what happened and how this relates to their memories, particularly when they experienced delirium (Murray et al., 2020). Very early childhood memories can be unclear, disjointed and primarily sensory, but reliving and updating can still be used. The content and mode of updating need to be appropriate and accessible to the age the person was at the time of the trauma. For example, a young child may need simple messages that they are safe and the trauma is not their fault. Imagery rescripting can be useful to help update the meanings linked to these memories. For example, their older self entering the scene to deliver these messages, comfort the child with a hug and take them to a place of safety (similar to the protocol described by Arntz & Weertman, 1999).

Updating a memory where the worst expected outcome has happened is possible. The purpose of cognitive work is to address excessively negative meanings, not to pretend that something bad has not happened. Sometimes there has been a tragic outcome to a trauma, like a death or permanent physical injury. Here, we look for associated personal meanings that keep the sense of current threat going like ‘the person is still suffering’, ‘I am alone forever without them’, ‘I let them down and did not do enough to prevent their death’, ‘my life no longer has a purpose’, or ‘people will find me disgusting’ and address meanings which are distorted. Similarly, with genuine responsibility or moral injury, we do not seek to absolve an individual of blame but to identify where beliefs have become distorted or generalised (e.g. ‘because I did this, I am a monster’ or ‘no-one can be trusted’). We also help people accept responsibility where it is due and consider with them how to move forward (Murray & Ehlers, 2021). Even when there is no clear updating of a meaning, we can try to help put a ‘time-tag’ on the memory to show that it is a ghost from the past rather than happening now as it feels. For example, a patient whose worst fear was that they would be raped, and this is what occurred, could still benefit from updating their memory with the reminder that ‘this is in the past, it is over, no one is touching me now’. As before, we would also check for associated meanings and update these as appropriate.

### 5 CT-PTSD is a talking therapy

Of course, talking is involved, but we think of CT-PTSD as a ‘doing’ therapy, as all good CBT should be. Experiential techniques are generally part of every session, whether that is reliving, creating written narratives, updating trauma memories, transforming images, practising stimulus discrimination, conducting surveys, behavioural experiments or a site visit. Between sessions, patients are engaged in reclaiming/rebuilding your life assignments, behavioural experiments and other tasks. Treatment should be active and involved and create new experiences that help change the threatening meanings of the trauma and its aftermath. A related misconception is that cognitive therapy only involves the verbal restructuring of cognitions. In fact, testing hypotheses experientially has been a cornerstone of cognitive therapy since its inception (Beck et al., 1979), and a wide range of techniques to address memories and behaviours, as well as beliefs, are important elements of CT-PTSD.

Our experience in supervision has been that surveys, out-of-office behavioural experiments and site visits are the least commonly used techniques, often relating to practical obstacles combined with therapist concerns about unexpected outcomes or patients experiencing flashbacks. We find surveys are powerful, particularly in addressing shame-related beliefs, and we encourage their regular use. As with reliving (Shearing et al., 2010), research has shown that site visits can be anxiety-provoking, but patients find them rewarding and beneficial (Murray et al., 2016), suggesting that some of these therapist concerns may be unfounded. Some therapists also report that they do not carry out site visits if patients have already revisited the scene of the trauma. We find site vists are helpful even when the trauma occurred in a much-visited place, including their own home, as patients are often using safety behaviours or avoiding some aspects of the experience and do not naturally use strategies during site visits that lead to new learning (piecing together the trauma memory, looking for new information, trigger discrimination, behavioural experiments). Video examples of how to carry out all the different techniques mentioned here, including various types of behavioural experiments can be found at www.oxcadatresources.com.

CT-PTSD also addresses the physical nature of PTSD. Re-experiencing symptoms are not only experienced in the cognitive domain; the whole body may re-experience the trauma. For example, patients may find themselves frozen in fear when a memory is triggered, or sweating, shaking, or vomiting, just as at the time of the trauma. It follows that our memory updates are not only verbal and cognitive, but physical as well. For example, for someone who was trapped and unable to move peri-traumatically, a verbal update ‘I can move now’ would be paired with physical movement during reliving, such as standing, waving the arms, and walking around. Talking alone may be insufficient for the physical elements of the trauma memory to be updated.

### 6 PTSD can’t be treated remotely

The COVID-19 pandemic forced many therapists to work remotely, sometimes for the first time, and perhaps to collect evidence against this misconception. The development and testing of internet-delivered trauma-focused PTSD treatments (including CT-PTSD; Ehlers et al., 2020, Wild et al., 2016) has shown preliminary evidence of effectiveness (Kuester et al., 2016), suggesting that remote delivery is possible. Wild et al. (2020) detailed how to carry out CT-PTSD remotely showing that, with some flexibility and creativity, all the usual treatment strategies are possible. For example, when practising trigger discrimination, therapists are encouraged to source possible triggers (audio and visual files) before the session, email them to the patient during the session, and listen or look at them together using a shared screen (if video-conferencing), listing the similarities and differences on a shared Word document. Behavioural experiments can be carried out with the patient over the phone. Site visits can be virtual, using Google Streetview, or with the patient attending the site (potentially with a trusted friend or family member) and speaking on the phone to the therapist. There are training materials about remote delivery available on www.oxcadatresources.com.

### 7 Dissociation rules out work on trauma memories

A common concern is that patients will dissociate during memory work, often leading therapists to avoid addressing trauma memories with patients who show signs of dissociation or first implementing long stabilisation phases. Research does not support the need for these changes. Hoeboer et al. (2020) and Halvorsen et al. (2014) found no evidence that pre-treatment dissociation moderates the effectiveness of trauma-focused treatments. In other words, techniques like memory updating still work when someone is prone to dissociation.

However, the experience of having a dissociative flashback is unpleasant for our patients and severe dissociation is likely to disrupt work on the trauma memories and prevent the integration of new information, so dissociation should be addressed in therapy. In CT-PTSD, patients are encouraged to identify and focus their attention on reminders of the ‘here and now’ to bring their awareness back into the room if they begin to dissociate. These might include objects with a positive association, photos, strong smells or tastes, or something to squeeze or hold. Having these handy is important during memory work for patients who dissociate, especially when working remotely. Moving around and hearing the therapist speak are also a useful reminders of the present. Trigger discrimination is taught and practised prior to work on the trauma memories for dissociative patients, alongside recognition and awareness of first signs of dissociation and its triggers.

Some adaptations to memory work may be appropriate with dissociation. For example, a written narrative is usually less immersive than imaginal reliving, so may be preferable, particularly when therapy is remote. The therapist can also reduce the intensity of reliving by asking the patient to keep their eyes open, speak in the past tense and by asking frequent questions. Continual movement can be helpful during memory work, such as walking on the spot, using a fidget spinner, or throwing a soft ball backwards and forwards, to keep the patient’s attention in the here and now and prevent freezing. The therapist can closely monitor the patient for signs of dissociation and intervene rapidly to bring their attention back into the present if needed.

A subgroup of patients with PTSD may experience long periods of dissociation (e.g. finding themselves in places without knowing how they got there). Careful identification of triggers (especially sensory triggers) and early signs of dissociation (e.g. starting to feel unreal) and use of the trigger discrimination technique with reminders of the here and now can help these patients stay in the present and thus reduce risk. Involvement of partners or family in helping patients spot triggers and signs of dissociation is helpful in early stages of therapy.

In summary, dissociation need not be a barrier to memory-focused interventions in CT-PTSD, nor is a long stabilisation phase required (see point 2). However, it is an important problem to address in therapy and some adaptations to trauma-focused techniques may be indicated.

### 8 PTSD is about fear

While early CBT models and studies of PTSD focussed on conditioned fear responses, TF-CBT programmes deal with the full range of negative emotions that can occur during and after traumatic events, including guilt, shame, anger, humiliation, betrayal, disgust, helplessness, hopelessness, horror and so on. Evidence suggests that only about half the emotions in the worst moments of traumatic memories are fear-based (Grey & Holmes, 2008). It follows that the meanings linked to these emotions are varied and idiosyncratic, so our approaches to understanding and addressing these meanings must also be personalised to each patient.

Often fear-based meanings like ‘I’m going to die’ are easy to spot, but we often find that further meanings are revealed if we ask follow-up questions like ‘what was the worst thing about that for you?’, or ‘what does that mean about you as a person?’. We try to avoid making assumptions about what might be distressing for an individual. For example, believing you are going to die is often associated with fear, but a further meaning may be uncovered like ‘I’m letting my family down by allowing this to happen’ associated with shame, or ‘I’m never going to see my children again’ associated with sadness. This is important because all of these different meanings will need to be updated to achieve a full emotional shift; not just ‘I survived, I’m alive’, but also ‘I didn’t allow this to happen, it wasn’t my fault’ and ‘I see my children every day’.

We also look for ways to consolidate these new meanings and not just in the verbal-cognitive domain; as described in point 5, trauma memories are also experienced physically and in imagery, so we update in those domains as well. For example, moving around to show that is now possible, looking at photos to consolidate an update like ‘I see my children every day’, touching a scar to show that a wound has now healed, re-reading survey results to consolidate a new meaning like ‘other people do not think I am to blame’, or accessing an image such a person who has died in a peaceful place to represent an update like ‘they are not in pain anymore’.

## Misconceptions about CT-PTSD

### 9 Exposure needs to be graded

Many therapists use graded exposure to approach trauma reminders and memories. This is a core technique in many trauma-focused treatments for PTSD, including Prolonged Exposure (Foa et al., 2007; Foa, & Rothbaum, 1998). It originated from behavioural models of phobias which emphasise achieving habituation to fear-provoking situations. In a cognitive approach to PTSD, the aim is to test trauma-related appraisals, which include beliefs of the likelihood of future harm or negative reactions of other people, so behavioural experiments are used to provide the best test of an appraisal. This approach can be less time-consuming than graded exposure. Rather than work through a hierarchy of feared situations, staying in each one long enough for anxiety to habituate, a test of the underlying belief (e.g. ‘I will be attacked again if I drop my guard in public’) via one or two behavioural experiments (e.g. going with the therapist to a busy shopping centre and deliberately dropping your guard by closing your eyes) may be sufficient to learn that the feared outcome does not occur. Of course, patients need to be willing to engage in an experiment, and support from the therapist (e.g. modelling the experiment first) is important, which is why we often do behavioural experiments during therapy sessions. Incidentally, the importance of testing feared predictions has also been highlighted in contemporary exposure therapies for PTSD as well (e.g. Craske et al., 2014).

Although we do not grade experiments, timing them is important. We usually find it helpful to do memory updating and trigger discrimination work first to reduce re-experiencing during challenging experiments and so that the ‘then versus now’ technique can be used during the experiment if memories are triggered. The latter is needed so that the patient is able to process that the predicted outcome did not occur during the experiment.

There is also a subtle difference in exposure to the trauma memory in different trauma-focused treatments. In CT-PTSD, the purpose of narrative writing or imaginal reliving is to access the important personal meanings associated with the worst moments of the trauma and then update these. Unlike prolonged exposure, where reliving is a major part of most sessions, this is usually required only two or three times in CT-PTSD, with more time spent addressing the meanings associated with the memory and generating updating information using cognitive interventions. Updating is started as soon as possible, rather than repeated detailed reliving sessions without updates.

### 10 CT-PTSD is protocolised and inflexible

We commonly hear this misconception, along with requests for a session-by-session protocol for delivering CT-PTSD. Therapist surveys of attitudes to protocols reveal mixed feelings; while many see the utility of empirically-derived guidance, others view them as restrictive and detrimental to the therapeutic relationship (Addis, & Krasnow, 2000). CT-PTSD is not a session-by-session protocol; it differs for every patient, depending on their individual formulation and needs. We appreciate that this can be challenging for therapists when they first learn the treatment. The core elements of treatment are similar for everyone, starting with psychoeducation, formulation, and reclaiming/rebuilding your life, before working on trauma memories and meanings, but there is a lot of flexibility about when and how to deliver certain treatment elements. For example, with a patient who experienced a sexual assault and feels very ashamed about the trauma, we may work first (before reliving the memory) on shame-related appraisals such as ‘people will think I let it happen because I didn’t fight back’ by introducing some psychoeducation about the freeze response and conducting a survey. This helps to provide potential updating information (e.g. ‘I didn’t fight back because I froze, which is a natural, instinctive reaction to threat. In the survey, no-one thought I had “let it happen” or blamed me.’) which can then be linked to the relevant hotspots in trauma memory in the first reliving or narrative writing session. For a patient who cannot sleep because lying in bed triggers disjointed, confusing memories of their trauma in hospital, we may use stimulus discrimination to address lying down as a trigger and develop a detailed timeline of their time in hospital to fill in the gaps in their memory, create a more coherent narrative, and identify hotspots which are being re-experienced. We may do a site visit early in treatment to help piece together the disjointed memory and to speak to hospital staff who can provide valuable information about what occurred.

In this way, we keep close to Ehlers and Clark’s (2000) cognitive model, always guided by the individual formulation and the patient’s goals, whilst delivering treatment adaptably and creatively. This approach is sometimes termed ‘flexibility within fidelity’ (Whittington & Grey, 2014).

## Summary and conclusions

Therapists can understandably hold beliefs about trauma-focused interventions which, amongst other factors such as organisational constraints, may lead us to hold back from certain techniques used in CT-PTSD and other trauma-focused cognitive behavioural therapies, particularly experiential work with trauma memories and out of the office behavioural experiments and site visits. This can limit the effectiveness of treatment and convey the message to patients that their problems are too severe or distressing to face. Often beliefs relate to an empathic wish to prevent distress or where therapists feel under-confident. In this paper, we have hopefully debunked some of the misconceptions which lead therapists to hold back or that may limit our flexibility and creativity. Within the framework of a collaborative and supportive therapeutic relationship, and sticking closely to the individual case formulation derived from Ehlers and Clark’s (2000) cognitive model, we encourage therapists to be curious, creative and confident in addressing the memories and meanings that are central to the experience of PTSD.

There will be other misconceptions about treatment that we all hold, and reflecting on our beliefs is an important part of ongoing professional development. Just as we encourage our patients to question their assumptions, and to explore and test their cognitions, we as therapists should do the same. For example, when we notice a feeling of reluctance about using a particular approach in treatment, we can reflect on our beliefs about it (alone and in supervision), consider evidence, and experiment with testing these out. Attending training is another valuable tool to broaden our knowledge, and evidence also shows it can lead to changes in therapists’ beliefs (Deacon et al., 2013), especially when experiential methods and case studies are included (Farrell et al., 2013).

Although we have focused here on individual level beliefs, we would also highlight the importance of systemic changes to reduce the research-practice gap. Some factors are difficult to change, such as the high demand for services combined with limited resources which impact on caseloads and session numbers. Others may be achievable, such as access to good quality training and structured, model-specific supervision. We also recommend the use of structured, focal assessments. We note that some IAPT services have developed specialist PTSD streams within their services staffed by therapists with a particular interest in PTSD and who receive additional training and supervision; we await evaluations of such initiatives with interest.

## Data Availability

No data are reported in this article.

## Key learning aims

- To recognise common misconceptions about trauma-focused CBT for PTSD and the evidence against them.
- To widen understanding of the application of cognitive therapy for PTSD (CT-PTSD) to a broad range of presentations.
- To increase confidence in the formulation-driven, flexible, active, and creative delivery of CT-PTSD.

## Key practice points

- Therapists may have beliefs about trauma-focused treatments that lead us to hold back from delivering potentially effective aspects of therapy. Recognising, researching, reflecting on and testing out our beliefs is an important part of developing as a therapist.
- Several misconceptions about trauma-focused CBT are common, such as that it is unsuitable for some types of trauma, cannot be delivered remotely and is inflexible, but available research and clinical guidance does not support these misconceptions.
- While sticking closely to evidence-based models of PTSD, and in collaboration with their patients, therapists can deliver treatment techniques flexibly and creatively, adjusting their practice to address a wide range of PTSD memories, meanings, and behaviours.
